# Ultrasound indicators of organ venous congestion: a narrative review

**DOI:** 10.1186/s13613-025-01609-x

**Published:** 2025-11-18

**Authors:** Zouheir Ibrahim Bitar, Ossama Sajeh Maadarani, Mohamad Bitar

**Affiliations:** 1 Critical Care Unit, Ahmadi Hospital, Kuwait Oil Company, PO Box 46468, 64015 Fahaheel, Kuwait; 2 Critical Care Medicine, Pulmonology Altru Hospital, Grand Forks, USA

**Keywords:** VExUS, Point of care ultrasound, Heart failure, Venous congestion

## Abstract

Acute kidney injury and other organ dysfunction in the setting of heart failure are primarily determined by a low cardiac output status and venous congestion, which is a sequence of increases in heart filling pressures. Early point-of-care ultrasound assessment of the inferior vena cava, lung ultrasound for pulmonary congestion, and focused echocardiography have become increasingly used in the bedside evaluation of congestive heart failure and assessment of the left ventricle. The congestion disrupts venous outflow in abdominal organs, most notably the kidneys and liver, and can be noninvasively evaluated with Doppler ultrasound, known as the venous excess. Such flow abnormalities have been repeatedly linked to congestive organ dysfunction and poorer clinical outcomes. In this review, we outline a thorough, bedside approach to assessing venous congestion using Doppler imaging. Venous Excess Ultrasound (VExUS) is an emerging protocol that offers a point-of-care ultrasonic method for grading systemic congestion and tailoring diuretic management. The purpose of this review is to evaluate VExUS's potential applications and critically appraise current evidence on its effectiveness in directing decongestive therapy for patients with acute decompensated heart failure. In conclusion, multiple Doppler venous congestion assessment emerges as a promising, noninvasive tool for the instantaneous assessment of organ congestion in cardiorenal syndrome, helping in the management of fluid and diuretic administration. Its accuracy, however, depends on the sonographer's proficiency. Larger-scale studies are needed to confirm their applicability in clinical practice

## Introduction

Point-of-care ultrasound (POCUS) is a well-established bedside examination for assessing cardiac function in various clinical situations, including low cardiac output states and venous congestion. POCUS includes assessing cardiac output, left ventricular filling pressures, ultrasonic lung B lines, and extravascular lung fluid with pleural effusion [1]. Incorporating lung ultrasound and cardiac ultrasound increases the diagnostic accuracy and assesses the severity of organ congestion in heart failure patients [1, 2]. Multiorgan congestion plays a crucial role in the pathophysiology of heart failure and cardiogenic shock, and organ-specific ultrasound evaluation of congestion and perfusion is vital [1, 2]. There is strong evidence that, independent of a reduction in cardiac output and renal blood flow in patients with heart failure, an epidemiological association exists with venous congestion and reduced glomerular filtration rate, as well as renal function tests [3, 4]. In acute heart failure, Mullens et al. [4] showed that higher CVP predicts worsening renal function in the hospital and does so to a greater extent than low cardiac index. The cardiac index was inversely associated with worsening renal function, although no association was observed with baseline glomerular filtration rate [4]. A 2-dimensional echocardiogram with Doppler should be initially performed in patients presenting with acute heart failure. This test is used to assess the function, size, wall thickness, and motions of the ventricles, as well as valve function [5–7]. Inferior vena cava (IVC) ultrasonic assessment and lung ultrasound (LUS) at the bedside using portable ultrasonic machines are recommended in the Consensus Statement of the Heart Failure Association of the European Society of Cardiology (ESC) as a comprehensive examination prior to discharge [8]. They provide reliable estimates of right atrial pressure and pulmonary congestion, respectively, and rapidly reflect changes in volume status in response to treatment.

LUS is important in detecting pulmonary congestion, but it does not evaluate the effect of congestion on the abdominal organs and cavities, which contribute significantly to deranged liver and kidney function in patients with right-sided heart failure [9]. Organ dysfunction in venous congestion is a sequence of increases in right atrial pressure, and importantly, pressure transmission to various abdominal organs and peripheral tissues [10]. Transmission of pressure alters the pattern of venous blood flow, primarily in the renal, portal, hepatic, and IVC veins, in a predictable manner, and these alterations can be quantified using venous Doppler [11, 12]. Using the venous Doppler can be an early warning sign for patients who are potentially at risk of organ dysfunction due to fluid overload or edema.

### Inferior vena cava (IVC)

Normal IVC diameter is associated with respiratory variability. In physiological conditions, the IVC diameter decreases and venous return increases during inspiration due to negative intrathoracic pressure, and it reverses during positive intrathoracic pressure [13]. Other factors associated with IVC diameter variation during the cardiac cycle, such as ventricular systole, also decrease in spontaneously breathing patients. Moreover, the position of critically ill patients affects the diameter of the IVC. However, the American Society of Echocardiography Guidelines recommended measuring IVC in the supine position [14].

Two important factors in critically ill patients should be determined: the IVC diameter and IVC variability. In case of acute RV failure, these can indicate right ventricular (RV) function in some clinical situations [15].

The IVC diameter assessment is often considered a noninvasive tool for measuring CVP [16]. Measuring CVP was described several decades ago [16] and has since become a standard method for assessing volume status and guiding intravenous fluid therapy. However, many subsequent studies indicate a poor association between CVP and blood volume and the inability of CVP and/or its changes to predict the hemodynamic response to a fluid challenge [1, 16]. Hence, relying on CVP for fluid management should not be the only deciding factor.

**Fig. 1:**
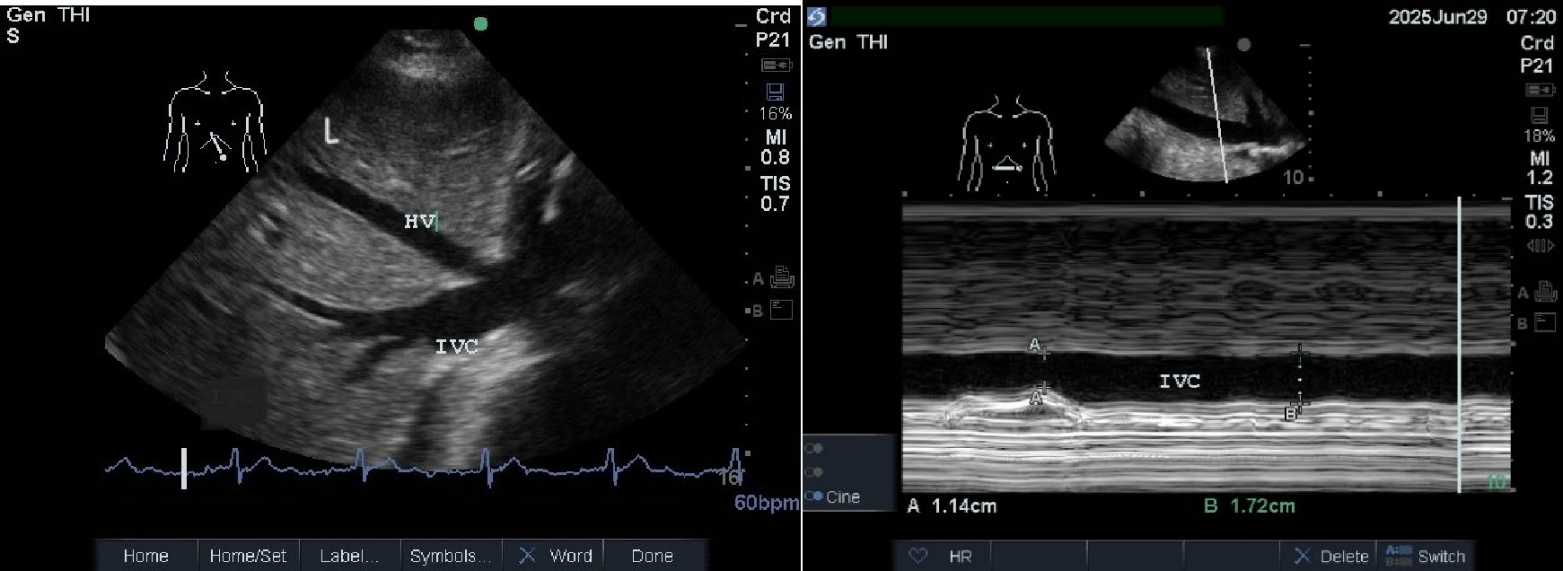
Ultrasound Epigastric view showing liver (L) with inferior vena cava (IVC ) and left hepatic vein (HV)

To evaluate the variation in the inferior vena cava (IVC) diameter during the respiratory cycle, we can use the IVC collapsibility index (IVCCI) (Fig. 1). The physician measures the maximum (IVCmax) and minimum IVC diameters (IVCmin) throughout this cycle. IVCCI is computed using the formula: (IVCmax - IVCmin) / IVCmax. In spontaneously breathing patients, an IVC diameter of less than 2.1 cm with IVCCI greater than 50%, accompanied by a sniff, usually indicates a central venous pressure (CVP) of 0 to 5 cmH2O. An IVC diameter of less than 2.1 cm and an IVCCI of less than 50% with a sniff suggests an elevated mean RAP 10 to 20 mm Hg cmH2O [17]. It is important to note that IVCCI has not been validated in patients requiring positive-pressure mechanical ventilation due to respiratory failure [18].

Intrathoracic pressure is one of the main determinants of IVC diameter. In a spontaneously breathing individual, during inspiration, the IVC diameter decreases as venous return increases. Aggravated IVC collapsibility may be observed in markedly negative intrathoracic pressure during forced inspiratory efforts in respiratory distress or exacerbation of chronic obstructive pulmonary disease, causing an increased venous return to the right atrium [19].

In ventilated patients, positive end-expiratory pressure may impede venous return during inspiration due to elevated intrathoracic pressure, thereby reducing the pressure gradient between the abdominal and thoracic compartments [8]. This pressure is transmitted to the right atrium and the IVC, which stretches in proportion to its compliance. Among patients with low cardiac reserve or those with preload dependence, such as those with severe hypovolemia, the IVC shows reduced compliance and limited distention, and its diameter may not vary [19, 20].

Elevated intra-abdominal pressure has a significant effect on both venous return, cardiac output, and the diameter of the IVC. Under these conditions, the IVC exhibits diminished compliance, which compromises its distensibility and responsiveness to changes in intravascular volume. This altered mechanical behavior can lead to misleading sonographic assessments, particularly in patients undergoing invasive mechanical ventilation, where standard IVC-based evaluations may yield false results regarding fluid status [18, 19].

Cardiac pathologies that impede venous return, such as right ventricular dysfunction, severe tricuspid regurgitation, and cardiac tamponade, elevate right atrial pressure, which in turn distends the IVC. The distended IVC reflects increased central venous pressure rather than intravascular volume status [19, 20].

While IVC measurements can estimate CVP and its changes, they are unreliable indicators of fluid responsiveness. A systematic review by Paul E Marik et al. revealed a weak correlation between CVP and blood volume and the inability of CVP/Delta CVP to predict the hemodynamic response to fluid administration. As a result, CVP should not be utilized to make clinical decisions about fluid management [15].

### Liver doppler waveforms

Changes in the hepatic vein (HV) and portal vein (PV) Doppler waves are the mainstay for assessing venous congestion and evaluating fluid tolerance. Each has its characteristics and waveform.

1. Hepatic vein Doppler. Although the right hepatic vein is conventionally favored for Doppler assessment due to its lateral orientation and consistent triphasic waveform, the left hepatic vein may offer superior Acoustic exposure with the subxiphoid approach [21]. A phased-array or curvilinear probe is used. Appropriate acoustic windows were used to image the hepatic veins using intercostal, subcostal, or transabdominal approaches. The left HV and middle HV are identified from a mid-subcostal or left lateral chest wall and an intercostal view. Hepatic venous waveforms are obtained by applying pulsed-wave Doppler at a depth of approximately 2–4 cm from the junction of the HV to the IVC. Incorporating an electrocardiogram trace is crucial for accurately interpreting hepatic vein (HV) Doppler waveforms. Without a simultaneous electrocardiogram, it’s easy to misinterpret the HV waveform.

**Fig. 2:**
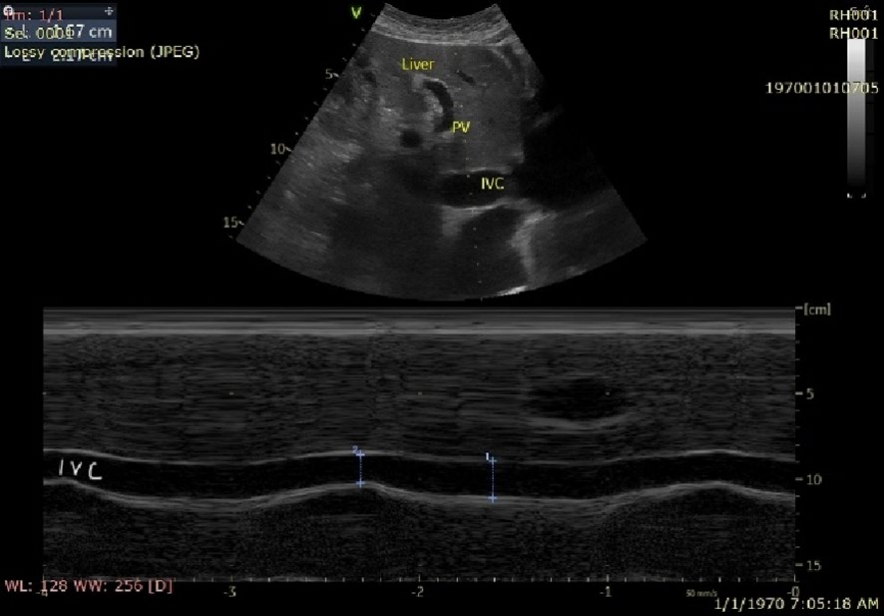
Ultrasound epigastric view showing the inferior vena cava (IVC) with M mode and respiratory variation; portal vein (PV)

**Fig. 3:**
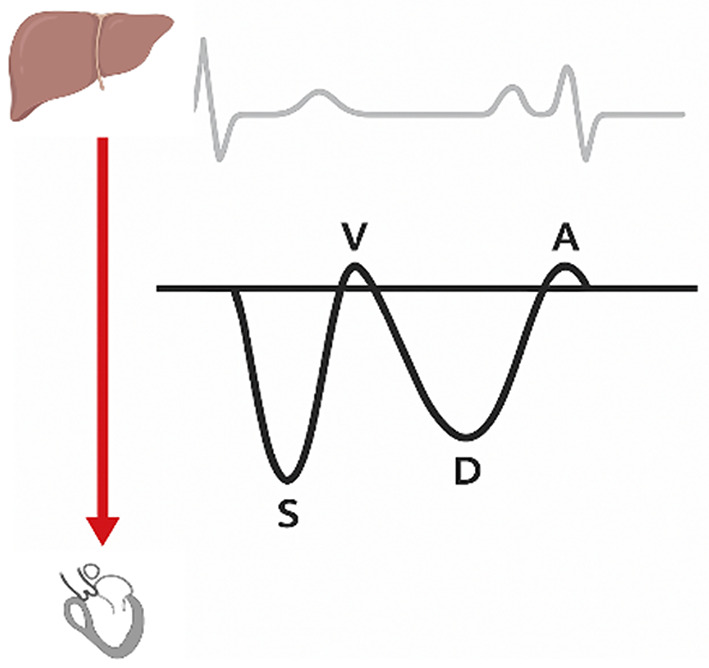
The Diagram shows the four waves in the normal spectral Doppler waveform of the hepatic vein

The normal hepatic vein waveform has four components (Figs. 2 and 3): a retrograde A wave, an antegrade S and D wave, and a transitional V wave (which may be antegrade, retrograde, or neutral) [13]. The A wave corresponds to atrial contraction. With the tricuspid valve open, blood is propelled in an antegrade direction toward the right ventricle and in a retrograde direction toward the IVC and into the hepatic veins, which gives the A wave on Doppler evaluation. The S wave represents ventricular systole. The initial component of the S wave is attributed to right atrial relaxation. The dominant and later component of the S wave is driven by ventricular systole. As the right ventricle contracts and ejects blood, the downward movement of the tricuspid valve annulus toward the apex creates a negative pressure gradient within the right atrium. This pressure differential, combined with the limited volume of the heart and pericardium, facilitates rapid blood flow from the hepatic veins into the right atrium, which is represented as the S wave on the Doppler tracing.

The V waves are generated by atrial filling. The height of the V wave can vary—it might be below, at, or above the baseline. This variation depends on whether there is continuous forward flow, a brief period of equilibrium with no flow, or a brief period of backward flow, respectively. In a healthy individual with the absence of venous congestion, the systolic wave (S-wave) exhibits a greater amplitude than the diastolic wave (D-wave), denoted as S >D. With the onset of mild congestion, a reversal in this relationship is observed, wherein the S-wave amplitude becomes less than that of the D-wave (S < D). In patients with severe congestive heart failure, the S-wave undergoes inversion (systolic reversal). The S wave appears above the baseline, while the D wave persists as the sole sub-baseline component. It is important to note that in instances of significant tricuspid regurgitation, this inverted S-wave signifies retrograde venous flow. Consequently, under these specific conditions, portal venous waveform evaluation becomes the preferred sonographic modality for volume assessment [22].

2) For Portal vein evaluation: A Curvilinear or phased array probe is used for Portal vein evaluation, and a transducer is placed at the posterior axillary line view between the ninth and eleventh intercostal space or midcoastal [22]. The portal vein is identified in the caudal part of the liver. Portal vein walls are more hyperechoic than hepatic veins. Flow wave characteristics are studied using pulsed-wave Doppler with the probe positioned in the middle of the vessel, and the waveform is obtained [23].

**Fig. 4:**
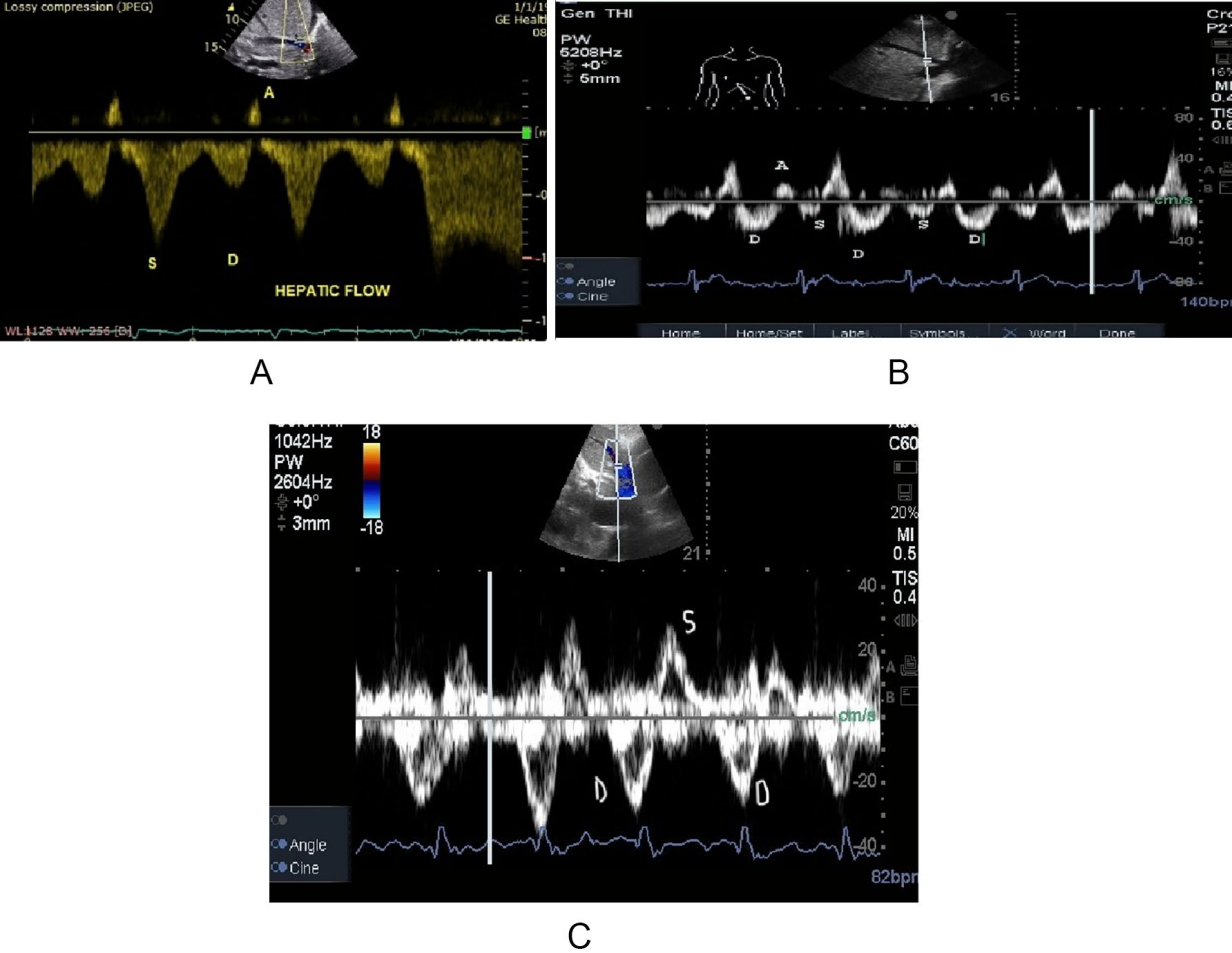
Hepatic vein Doppler waves progression;** A** normal S > D;** B** D > S; **C** systolic reversal

**Fig. 5:**
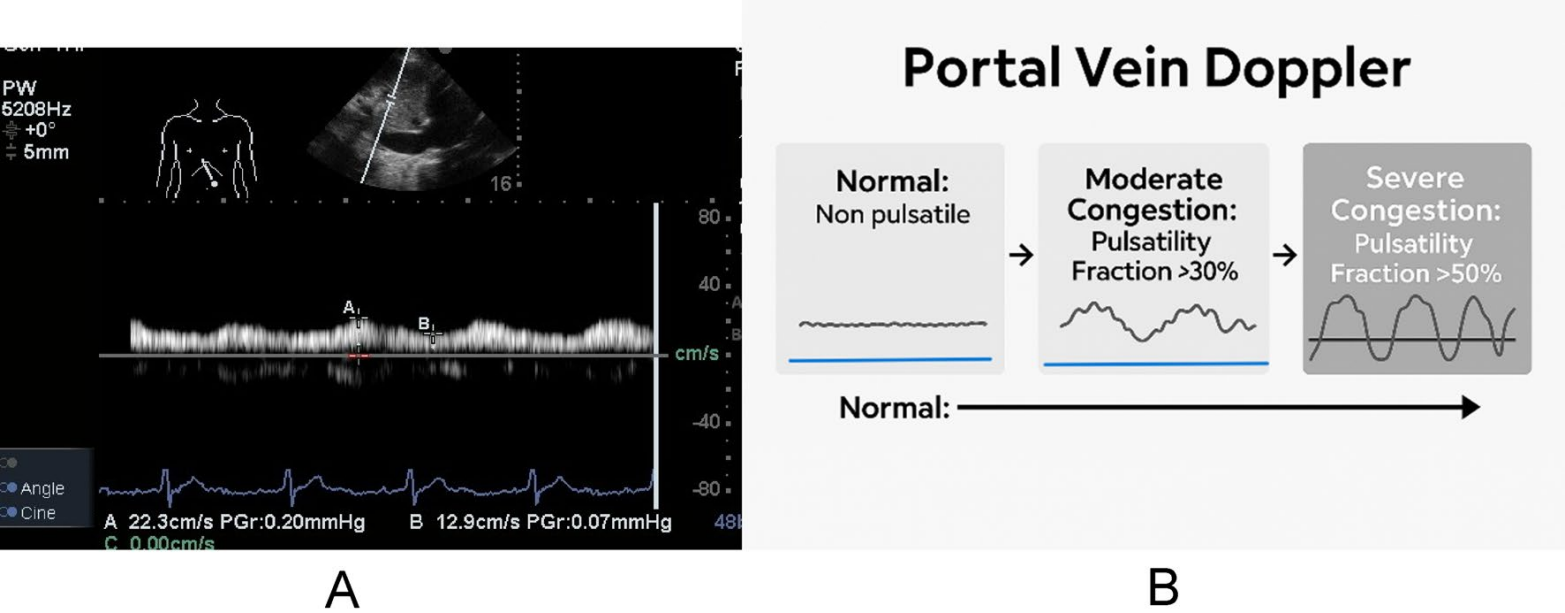
**A** portal vein flow waveform with pulsatility index 42%;** B** alterations occurring with progressive venous congestion

The Portal vein (PV) flow is low-velocity (10 to 30 cm/s), usually monophasic, but can be biphasic (Fig. 4). The PV waveforms are directed toward the transducer with minor variations throughout the cardiac cycle, although respiratory variation can be observed. The portal pulsatility index is calculated as (V. maximum – V. minimum) / V. maximum × 100%, where V is velocity. The PV is abnormal if the pulsatility index exceeds 30% (Fig. 5). Furthermore, the pulsatility index (PPI) was evaluated as a continuous variable to determine if subtle changes in portal vein flow could serve as significant indicators of venous congestion [23].

With reduced systolic flow in mild congestion and increased congestion, pulsatility increases. The systolic flow disappears in severe liver congestion. In more severe cases, the flow can reverse to retrograde (flow directed away from the transducer) (Fig. 4).

Measuring the PV pulsatility index quantifies flow variation throughout the cardiac cycle and indicates liver congestion. An index of 30% highly suggests liver congestion and is associated with a higher incidence of adverse kidney outcomes (odds ratio, 2.2; 95% CI, 1.3–3.6) [24].

###  Intra-renal venous doppler

**Fig. 6:**
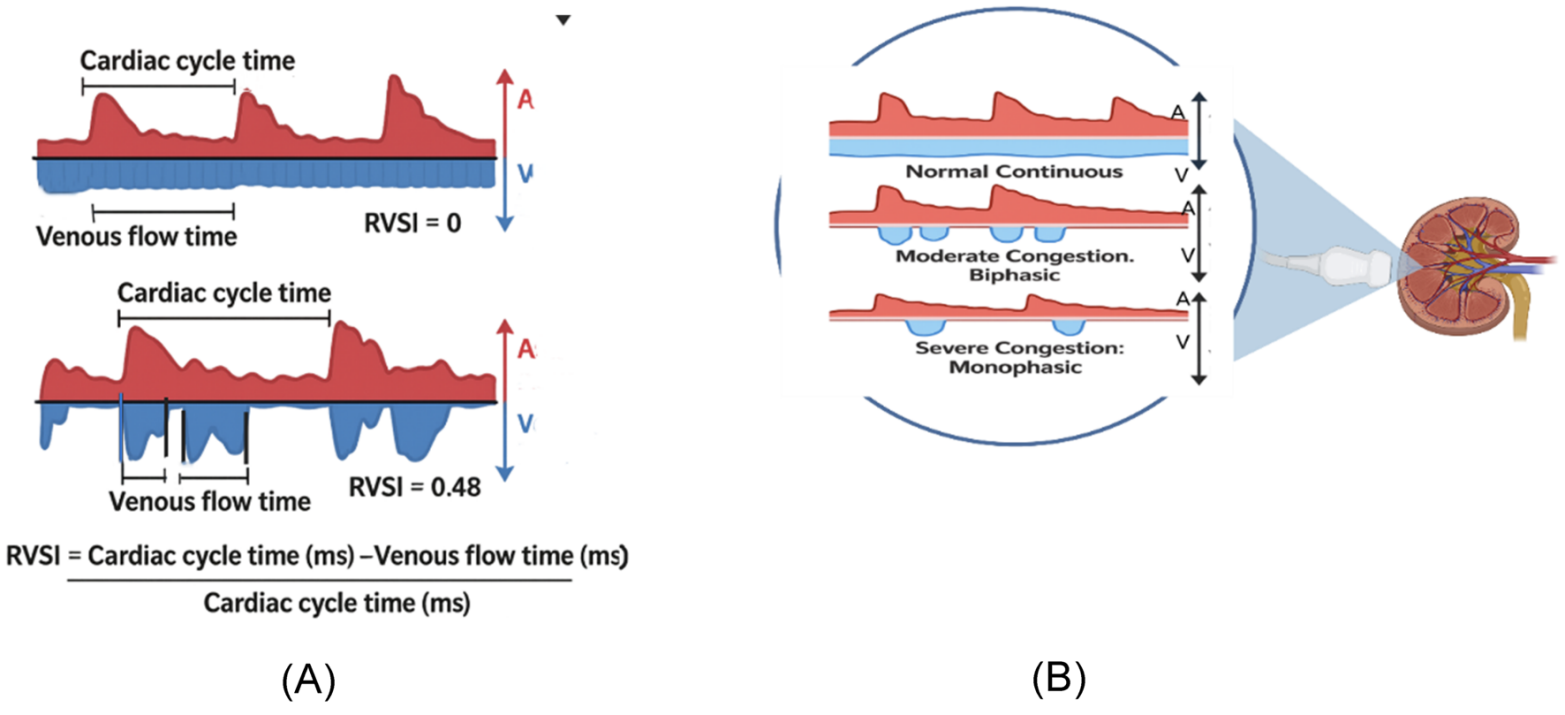
**A** Normal intrarenal Doppler venous flow waves and progression occurring with venous congestion.** B** Quantification of intrarenal venous flow using the renal venous stasis index (RVSI). Higher RVSI values are observed with worsening degrees of venous congestion. A, arterial flow; V, venous flow

Intra-renal Doppler ultrasonography images can be obtained in the supine position, using the lateral costal window, with a color Doppler velocity range of approximately 16 cm/s and placing the pulse Doppler over the interlobar vessels [25]. The waveform is usually considered adequate when both arterial (above the baseline) and venous (below the baseline) components are visible for two or more cardiac cycles (Fig. 6a). Intra-renal venous Doppler normally has a continuous monophasic flow below the baseline, progressively becoming interrupted with two phases, analogous to the S and the D waves of the hepatic vein flow. Similar to the hepatic vein pattern, venous congestion worsens, the S wave becomes smaller, and the D wave becomes more pronounced. Eventually, the S wave disappears entirely, leaving only a monophasic D wave. The intra-renal vein Doppler waveforms were considered abnormal if a biphasic or monophasic renal vein flow pattern was present. This was defined as discontinuous venous flow with either a systolic/diastolic or diastolic-only pattern ( 27).

An important parameter derived from renal venous Doppler is intrarenal venous flow (IRVF). The IRVF pattern is a novel indicator for determining renal congestion or overload in the absence of obstructed venous flow. As congestion progresses, a discontinuous flow pattern will emerge. The waveform changes are categorized into four flow patterns: continuous, pulsatile, discontinuous, biphasic discontinuous (characterized by venous peaks during both systole and diastole), and monophasic discontinuous (with a venous peak during diastole) (Fig. 3). The changes in the renal venous Doppler were quantified using an index of venous flow time to cardiac cycle time, called the renal venous stasis index (RVSI) [26]. The RVSI is a novel continuous ratio that quantifies the proportion of the cardiac cycle in which no renal venous outlet flow occurs. It is calculated using the following formula: (cardiac cycle time-venous flow time)/cardiac cycle time [26] (Fig. 6b).

A monophasic pattern and severe alteration of IVRF, observed during a Doppler ultrasound, can be a marker of venous congestion in patients with right-sided heart failure, especially following cardiac surgery [27]. The monophasic waves were independently linked to acute kidney injury [27]. The IRVF pattern exhibits a strong correlation with venous congestion following cardiac surgery. The presence of a discontinuous pattern of IRVF showed the occurrence of renal congestion as well as a poor prognosis.

In patients admitted with acute heart failure and the early post-discharge period, the congestive pattern IRVF was associated with a higher increase in serum creatinine values [26]. In cases of ongoing renal congestion, patients with pre-existing kidney dysfunction exhibited significantly higher levels of creatinine both at baseline and 90 days post-discharge. However, a positive response to diuretics and/or improvements in decongestion markers mitigated this correlation [27].

Alternative Doppler Techniques for Assessing Venous Congestion.

#### Femoral venous doppler (FVD)

The femoral vein (FV) is evaluated using a high-frequency linear probe (10–14 MHz). The linear probe is placed approximately one cm below the inguinal ligament, parallel to the ligament, with the linear probe marker facing cranially. The short axis of the femoral artery vein will be localized, followed by turning the probe 90°, and the longitudinal axis of the FV will be examined. The FV is examined, in supine position, by two-dimensional, pulsed-wave Doppler and color flow Doppler [28].

The FVD waves are described as normal, pulsatile, or pulsatile with flow reversal. Pulsatile and pulsatile with flow reversal FVD patterns were considered suggestive of venous congestion ( 29).

Many trials were conducted to evaluate the FV as a surrogate for IVC. Regarding the collapsibility index (CI), during ultrasound evaluations, the IVC CI did not correlate with the subclavian vein CI, the internal jugular venous CI, or the FV CI in volume-overloaded patients. It was concluded that superficial venous vessels cannot be used as an alternative to the IVC. The authors suggested that the lack of correlation might be due to compression of the veins during ultrasound measurements [29]. Venous congestion was assessed in adult post-cardiac surgery patients using the venous excess ultrasound (VExUS) score and FVD. The accuracy of VExUS and FVD for detecting venous congestion was 80.37 (95% CI: 71.5 to 87.4) and 74.7 (95% CI: 65.4 to 82.6), respectively. FVD shows moderate agreement with VExUS grading and may be a simple, valuable tool for assessing venous congestion [30]. However, FVD is less reliable in cases of high intra-abdominal pressure, cirrhosis, and respiratory distress. It is not valid in deep venous thrombosis and produces confusing results in varicose veins with saphenofemoral junction incompetence [30].

####  Internal jugular vein ( IJV)

The IJV is a superficial vessel, easily compressible, located close to the carotid artery, with a course under the sternocleidomastoid muscle, and can be easily detected by ultrasound. The patient is assessed in a semi-recumbent position, with neck elevation at 30–45 degrees. A high-frequency linear ultrasound transducer (∼10 MHz) is used, placed just below the angle of the jaw (around 5 cm), in the area of the sternocleidomastoid muscle. In addition to the B-mode, the IJV diameter and its dynamic changes, including during a Valsalva maneuver, can be measured using M-mode ultrasound.

**Fig. 7:**
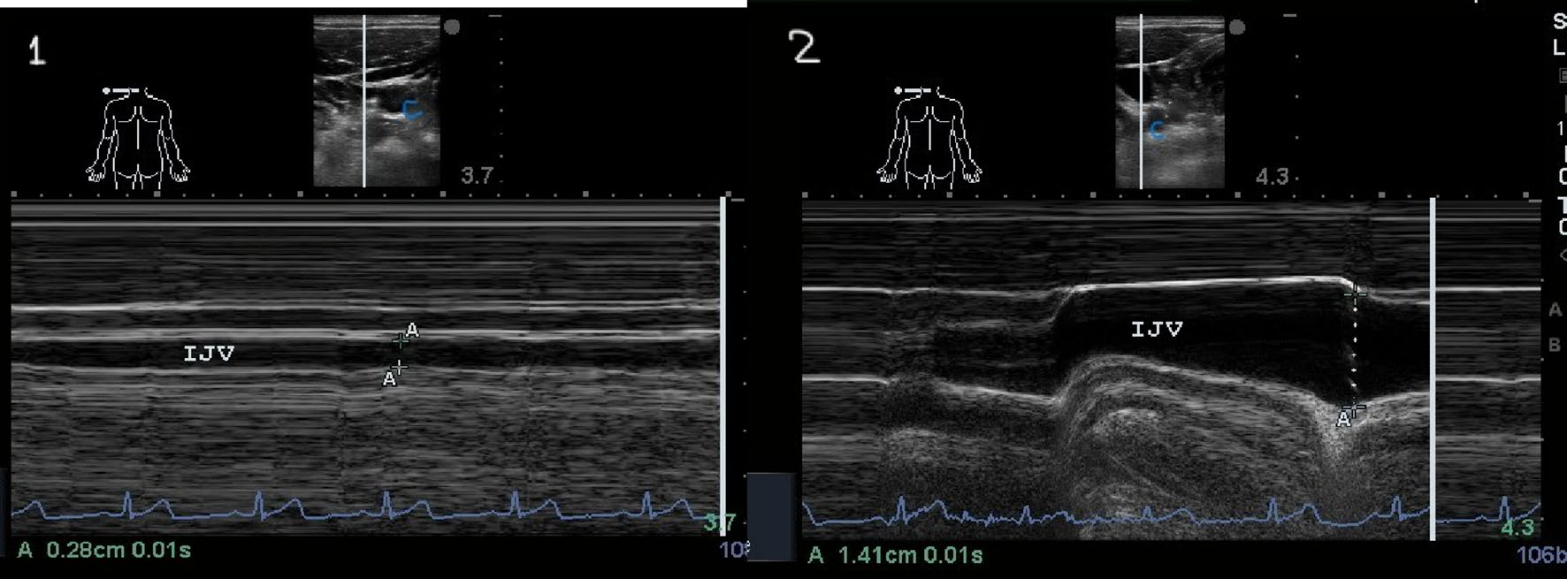
Jugular vein Measurements. The internal jugular vein (IJV) is identified, and JV diameter (JVD) and its changes are measured continuously by M-mode ultrasound using a linear high-frequency probe (10 MHz) [1] at rest in the expiratory phase, 0.28 cm [1], then during a Valsalva manoeuvre (1.41 cm). The ratio between the maximum JV diameter during Valsalva and the diameter at rest (JVD ratio) is calculated and is equal to 5 (normal)

**Fig. 8:**
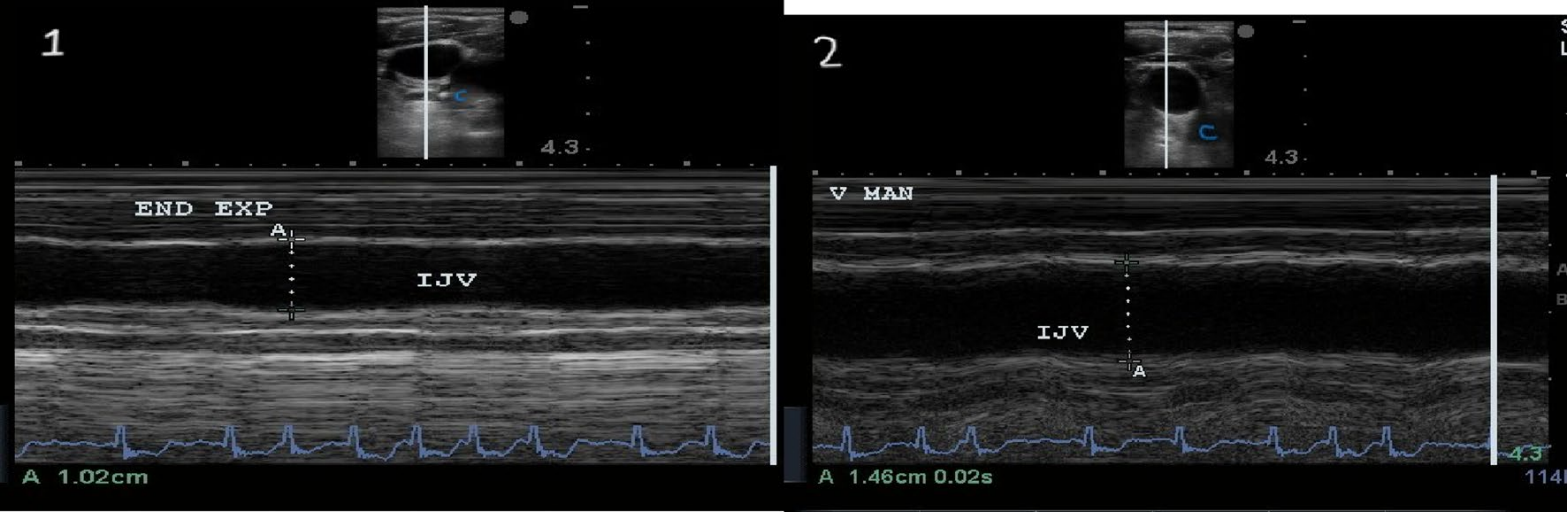
[1] End exp, end expiration; [2] V MAN, Valsalva manoeuvre, JVD ratio is 1.48 (less than 2) in a patient with acute heart failure

The Jugular Vein Diameter Ratio (JDVR) is the ratio between the maximal diameter during the Valsalva manoeuvre and at the end of the expiratory phase (Fig. 7). In healthy people and in patients with adequately controlled congestion, the IJV diameter is small at rest (0.10–0.15 cm). The Valsalva maneuver increases the IJV diameter to its maximum (approximately 1 cm) in individuals with and without intravascular congestion, due to limited vessel compliance (+ 4, + 5). For patients without congestion, the JVD R is ≥ 4. Decreased JVD ratio to < 2 in cases of worsened intravascular congestion, due to an increase in the IJV diameter at rest [31, 32] (Fig. 8). Another study used changes in cross-sectional area during the Valsalva maneuver [33]. A positive test indication of high right atrial pressure and hypervolemia in heart failure was defined as an increase of < 66% in right IJV cross-sectional area during Valsalva, with improvement in congestion when the cross-sectional area reached ≥ 66% [33].

In a systematic review, the JDVR revealed no evidence of good interrater and intra-rater reliability [34]. In ambulatory patients with heart failure, a low JVDR can identify patients with higher plasma NT-proBNP levels, correlates with invasive right atrial pressure measurement, and right ventricular dysfunction [31, 33]. The IJV ultrasound examination could be used when the IVC ultrasound is not feasible because of poor acoustic windows in case of obesity, severe bowel distension, and surgical wounds [34]. Physicians should be cautious in the interpretation of IJV ultrasound in certain cases of chronic pulmonary hypertension, pulmonary embolism, or cardiac tamponade, where hypovolemia can coexist with increased IJV diameter [34].

### The venous excess ultrasound score

The Venous Excess in Ultrasound Score (VExUS) is a point-of-care ultrasound (POCUS) grading system developed by Beaubien-Souligny et al. in 2020 to quantify the severity of venous congestion ( 36). Initially, the system was designed to predict the occurrence of acute kidney injury (AKI) in patients who had undergone cardiac surgery. VExUS combines measurements of the inferior vena cava (IVC) diameter with Doppler flow patterns of the hepatic vein, portal vein, and renal interlobar vein to assess and grade the severity of venous congestion [35].

In patients with right heart failure, the combination of bedside hepatic and portal vein Doppler ultrasounds, performed by POCUS-trained clinicians, is a valuable tool for assessing venous hypertension [36]. This readily available technique can holistically enhance hemodynamic profiling and inform direct therapeutic strategies [37].

For cardiac surgery patients, venous excess ultrasound (VExUS) has been established as a grading system to assess venous congestion and predict acute kidney injury [27]. This study identified severe congestion as having the strongest correlation with the development of subsequent acute kidney injury (AKI) compared to other combinations of ultrasonographic features. Significant flow abnormalities in multiple Doppler patterns characterize severe congestion. The Severe VExUS grade is defined by a dilated inferior vena cava (IVC) (≥ 2 cm) combined with at least two severe abnormalities.

**Fig. 9:**
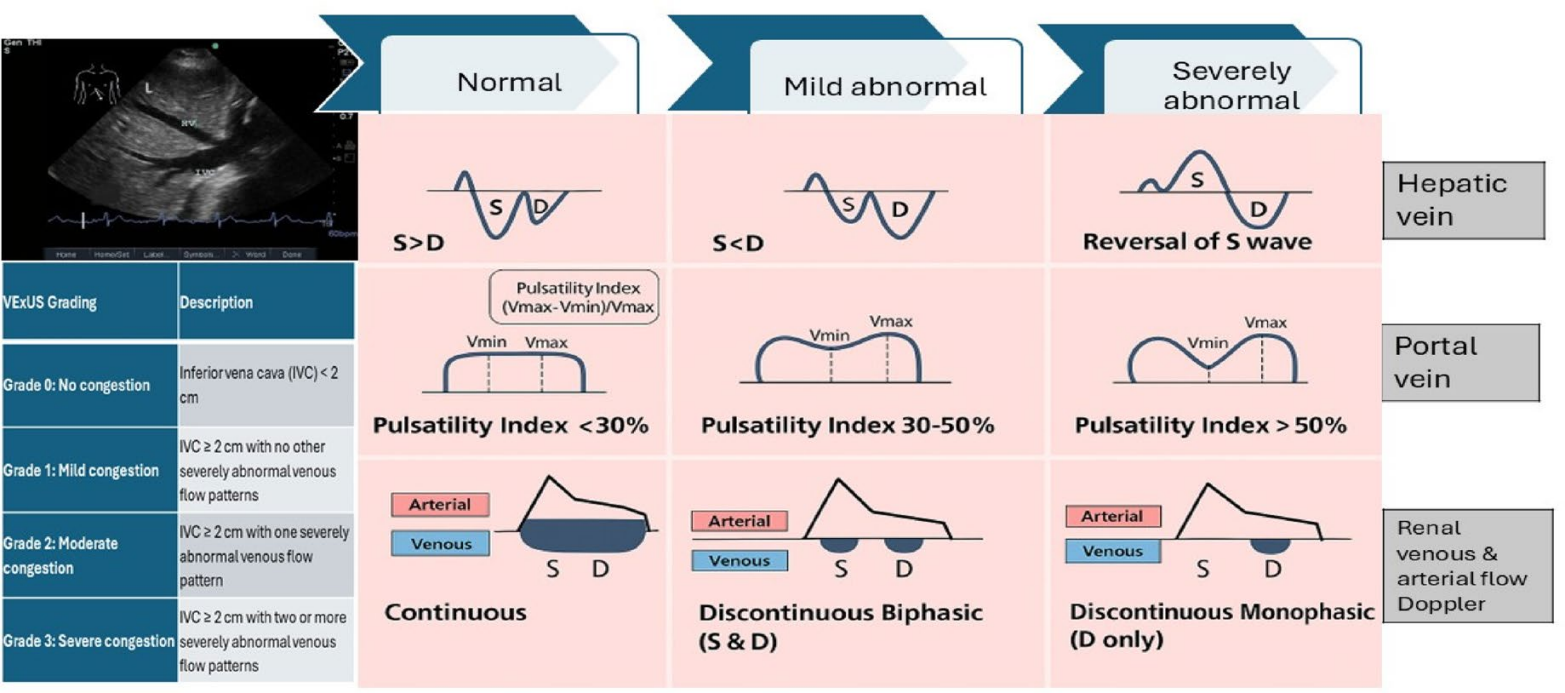
Components of the Venous Excess Ultrasound Grading System (VExUS) scoring system and grading. S– systole; D– diastole; L liver ; HV, hepatic vein; IVC, inferior vena cava

The VExUS grading system first assesses IVC, followed by hepatic, portal, and intrarenal veins. Venous Doppler flow waveforms are categorized as normal, mildly abnormal, or severely abnormal (Fig. 9) [27].

The conceptual review, employing the grading system of Rola et al., assesses the presence of a plethoric inferior vena cava and individually evaluates the hepatic, portal, and renal veins for normal, mild, or severe congestion. Based on these assessments, the VExUS grade is assigned as follows: grade 0 (no congestion), grade 1 (mild congestion only), grade 2 (severe congestion in one organ), and grade 3 (severe congestion in at least two of the three organ systems). (Fig. 9) [37]. Therefore, the present study introduced a new tool for investigating the pathophysiology of cardiorenal syndromes by directly measuring intrarenal vein pressure rather than through CVP. Discontinuous IRVF, particularly the monophasic pattern and the aggravated VExUS grading system, may provide additional information to comprehensively evaluate venous congestion or fluid status and provide guidance for timely fluid removal or discontinued fluid resuscitation.

### VExUS and diuretic response

There may be a relationship between the VExUS score and the effectiveness of diuretics during hospitalization for heart failure. A small cohort study demonstrated a lower diuretic effect in patients with high VExUS grades compared to those with grade 0 and I VExUS scores, independent of admission creatinine level and prior use of loop diuretics [38]. The RVSI had the best ability to predict low diuretic efficiency among the venous congestion assessment parameters (AUROC: 0.76 (0.60; 091) *p* = 0.001) [38]. Patients with acute kidney injury who required a high dose of furosemide in grade >1 and who had improved VexUS score over 48 h showed an increase in the number of renal replacement therapy (RRT)-free days in 28 days [39]. In patients hospitalized with acute coronary syndrome, it was found that each increasing degree of VExUS, a higher proportion of patients developed AKI: VExUS = 0 (10.8%), VExUS = 1 (23.8%), VExUS = 2 (75.0%), and VExUS = 3 (100%; *P* < 0.001). A significant association between VExUS ≥ 1 and AKI was found [odds ratio (OR): 6.75, 95% confidence interval (CI): 2.21–23.7, *P* = 0.001] [40]. After multivariable analysis, only VExUS ≥ 1 (OR: 6.15; 95% CI: 1.26–29.94, *P* = 0.02) remained significantly associated with AKI. VExUS is a strong predictor of AKI, beyond hemodynamic parameters and intravenous contrast dose [40]. In the Prospective Evaluation of Venous Excess Ultrasound for Estimation of Venous Congestion, where 81 patients underwent right heart catheterization, this study suggests that the VExUS ultrasound technique may accurately detect venous congestion, aligning with measurements from right heart catheterization (RHC) in specific heart patients undergoing planned RHC. Higher VExUS grades effectively identified elevated right atrial pressure (RAP) compared to standard bedside assessments. Additionally, increased VExUS grades were linked to acute kidney injury (AKI) in hospitalized individuals, supporting previous findings that VExUS can indicate kidney vein hypertension associated with AKI. The observed link between VExUS and weight loss in heart failure patients on diuretics suggests that VExUS might predict diuretic response and resistance. While further research is needed, the results indicate that VExUS could improve diagnostic and treatment abilities at the bedside and enhance our understanding of venous congestion. Future studies should further investigate VExUS [41].

### Right-sided heart failure and pulmonary hypertension

The most reliable and frequent tests for assessing pulmonary hypertension and venous congestion remain right heart catheterization (RHC) and the controversial central venous pressure, with direct assessment of RAP and pulmonary capillary wedge pressure [41].

In the same prospective study, Longino et al. assessed the correlation of VExUS grade with RAP compared with IVC diameter in patients undergoing RHC. They identified a significant positive correlation between RAP and VExUS grade (*p* < 0.001; R2 = 0.68) [42]. The VExUS scoring system exhibited a noteworthy capacity to predict a right atrial pressure (RAP) of 10 mmHg or higher, as indicated by a substantial area under the receiver operating characteristic curve (AUC) of 0.99 (95% confidence interval [CI] 0.96–1). This predictive accuracy surpassed isolated inferior vena cava (IVC) diameter measurements, yielding an AUC of 0.79 (95% CI 0.65–0.92). In essence, VExUS demonstrated a greater ability to identify elevated RAP values than did IVC diameter alone or its collapsibility index [41].

Research involving 124 patients with heart failure supports these observations. That research concluded that evaluating how the right internal jugular vein changes with respiration, along with measurements of the inferior vena cava’s size and its response to breathing, enhances the precision of non-invasive determination of right atrial filling pressure [42]. This study introduces a basic 3-point scale that uses bedside ultrasound to effectively gauge fluid status in heart failure patients. This scale incorporates the absence of respiratory variation in the right internal jugular vein, an end-expiratory inferior vena cava diameter of 21 mm or greater, and the lack of respiratory collapse in the inferior vena cava. The scale demonstrated strong efficacy in identifying elevated right atrial pressure (≥ 10 mm Hg) and was more effective than relying solely on inferior vena cava characteristics. In over two-thirds of the patients, the filling pressures on the right and left sides of the heart were consistent, and the scale showed a weak relationship with pulmonary capillary wedge pressure.

This approach offers a straightforward and easily implemented method for treating heart failure patients [42].

### VExUS as a prognosis predictor

Worsening of renal function was noticed in patients with acute heart failure in VExUS grade 3. It showed a higher incidence of diuretic resistance, a need for inotropic and/or vasopressor support, and, during the hospital stay, a worse prognosis [43, 44]. In A total of 125 patients with severe AKI, readily measured ultrasonographic markers of congestion are associated with higher mortality, although an adverse impact on kidney recovery was not observed. Although not significantly associated with major adverse kidney events at 30 days [45]. Further study is needed to determine whether fluid management strategies guided by point-of-care ultrasound impact clinical outcomes [45].

## Ongoing research and future directions

One study aims to determine the link between venous congestion detected by Doppler ultrasound and the necessity for renal replacement therapy (RRT) or mortality in patients experiencing septic shock. This research is a sub-study of the ANDROMEDA-SHOCK 2 trial, a randomized controlled trial (RCT) evaluating hemodynamic resuscitation in septic shock. The study plans to enroll at least 350 adult patients within 4 h of meeting the Sepsis-3 criteria for septic shock. The primary outcome measured will be RRT or death within 28 days of septic shock [46].

Another study proposes that using a noninvasive ultrasound protocol in septic patients, combining a modified lung ultrasound score with the VExUS protocol (VExLUS), could enhance the detection of fluid overload and aid clinicians in decision-making regarding fluid therapy [47]. The study’s main secondary objectives are to assess if there is a link between different VExLUS grades and the negative effects of giving many fluids, find out if there is a connection between signs of congestion in kidney ultrasounds and the development or worsening of acute kidney injury and to explore if higher VExLUS score are related to longer hospital stays and higher mortality rates [47].

## Limitations

Patient-specific factors can hinder the effectiveness of multiorgan ultrasound. Conditions frequently seen in critically ill patients, such as excess body fat, widespread edema, and the presence of medical devices, can significantly reduce the accuracy and practicality of this technique. These factors can compromise the clarity of ultrasound images, making it challenging to obtain reliable assessments. VExUS requires specialized expertise, and it should be acknowledged that venous congestion studies assess organ afterload. VExUS should be evaluated in the context of the patient’s general condition. The pulsatility index and mean velocity of portal vein blood flow decrease as hepatic fatty infiltration severity increases [48]. Otherwise, in patients with liver cirrhosis, the arterial pulsatility index was significantly higher than in controls and directly correlated with the hepatic venous pressure gradient [49]. Patients with severe tricuspid regurgitation may have a systolic reversal in the hepatic vein Doppler, even if cardiac filling pressures are normal or low [50]. Using VExUS without a thorough grasp of its technical limitations can lead to clinical misjudgments.

## Conclusion

Venous congestion is a crucial mechanism in the pathophysiology of renal failure in various diseases that affect the heart and kidneys. Venous Doppler examination and assessment of different organs is the only available bedside tool for assessing multiple organ congestion, which may guide clinicians in managing and evaluating intravascular volume status in various medical situations. The VexUS has a reasonable prognostic value. While there is a current scarcity of rigorously controlled intervention studies confirming the impact of a strategy employing this method, compelling logic suggests that an approach integrating point-of-care ultrasound could offer distinct advantages over current practices, which often exhibit considerable inconsistency among healthcare professionals.

## References

[1] Soliman Aboumarie H, Tavazzi G, Via G, et al. Cardiac ultrasound in cardiovascular emergency and critical care. A clinical consensus statement of the European association of cardiovascular imaging (EACVI), the acute cardiovascular care association (ACVC) of the ESC, and the European association of cardiothoracic anaesthesia and intensive care (EACTAIC). *Eur heart J cardiovasc imaging*. Published Online August. 2025;21. 10.1093/ehjci/jeaf246. 10.1093/ehjci/jeaf246 40838793

[2] Mullens W, Damman K, Harjola VP, et al. The use of diuretics in heart failure with congestion — a position statement from the heart failure association of the European society of cardiology. Eur J Heart Fail. 2019;21(2):137–55. 30600580 10.1002/ejhf.1369

[3] Damman K, Navis G, Smilde TD, Voors AA, van der Bij W, van Veldhuisen DJ, Hillege HL. Decreased cardiac output, venous congestion and the association with renal impairment in patients with cardiac dysfunction. Eur J Heart Fail. 2007;9:872–8. 17586090 10.1016/j.ejheart.2007.05.010

[4] Mullens W, Abrahams Z, Francis GS, Sokos G, Taylor DO, Starling RC, Young JB, Tang WH. Importance of venous congestion for worsening of renal function in advanced decompensated heart failure. J Am Coll Cardiol. 2009;53:589–96. 19215833 10.1016/j.jacc.2008.05.068 PMC2856960

[5] Yancy CW, Jessup M, Bozkurt B, American College of Cardiology Foundation; American Heart Association Task Force on Practice Guidelines, et al. 2013 ACCF/AHA guideline for the management of heart failure. Circulation. 2013;128:240–327. 10.1161/CIR.0b013e31829e8776 23741058

[6] Ponikowski P, Voors AA, Anker SD et al. (2016) ESC guidelines for the diagnosis and treatment of acute and chronic heart failure: the task force for the diagnosis and treatment of acute and chronic heart failure of the European Society of Cardiology (ESC). Developed with the special contribution of the Heart Failure Association (HFA) of the ESC. Eur Heart J 2016; 37: 2129–2200. 10.1093/eurheartj/ehw128 27206819

[7] Martens P, Nijst P, Mullens W. Current approach to decongestive therapy in acute heart failure. Curr Heart Fail Rep. 2015;12:367–78. 26486631 10.1007/s11897-015-0273-5

[8] Metra M, Adamo M, Tomasoni D et al. Pre-discharge and early post-discharge management of patients hospitalized for acute heart failure: A scientific statement by the Heart Failure Association of the ESC. Eur J Heart Fail. 2023 May 18. 10.1002/ejhf.2888. Epub ahead of print. PMID: 37448210. 10.1002/ejhf.2888 37448210

[9] Verbrugge FH, Dupont M, Steels P, et al. Abdominal contributions to cardiorenal dysfunction in congestive heart failure. J Am Coll Car Diol. 2013;62(6):485–95. 10.1016/j.jacc.2013.04.070 23747781

[10] Jessup M, Costanzo MR. The cardiorenal syndrome: do we need a change of strategy or a change of tactics? J Am Coll Cardiol. 2009;53(7):597–9. 19215834 10.1016/j.jacc.2008.11.012

[11] Tang WHW, Kitai T. Intrarenal venous flow: a window into the congestive kidney failure phenotype of heart failure? JACC Heart Fail. 2016;4(8):683–6. 27395345 10.1016/j.jchf.2016.05.009

[12] Smith HJ, Grøttum P, Simonsen S. Ultrasonic assessment of abdominal venous return. II. Volume blood flow in the inferior Vena Cava and portal vein. Acta Radiol Diagn (Stockh). 1986;27(1):23–7. 3515855 10.1177/028418518602700105

[13] Barbier C, Loubieres Y, Schmit C, Hayon J, Ric^ ome JL, Jardin F, Vieillard-Baron A. Respiratory changes in inferior Vena Cava diameter are helpful in predicting fluid responsiveness in ventilated septic patients. Intensive Care Med 30: 1740–6, 2004 15034650. 10.1007/s00134-004-2259-8 15034650

[14] Beigel R, Cercek B, Luo H, Siegel RJ. Noninvasive evaluation of right atrial pressure. J Am Soc Echocardiogr 26: 1033–42, 2013 23860098 26. 10.1016/j.echo.2013.06.004 23860098

[15] Marik PE, Baram M, Vahid B. Does central venous pressure predict fluid responsiveness? A systematic review of the literature and the Tale of seven mares. Chest. 2008;134:172–8. 18628220 10.1378/chest.07-2331

[16] Ciozda W, Kedan I, Kehl DW, Zimmer R, Khandwalla R, Kimchi A. The efficacy of sonographic measurement of inferior Vena Cava diameter as an estimate of central venous pressure. Cardiovasc Ultrasound. 2016;14(1):33. 10.1186/s12947-016-0076-1. 27542597 10.1186/s12947-016-0076-1 PMC4992235

[17] Prekker ME, Scott NL, Hart D, Sprenkle MD, Leatherman JW. Point-of-care ultrasound to estimate central venous pressure: A comparison of three techniques. Crit Care Med 41: 833–41, 2013 23318493. 10.1097/CCM.0b013e31827466b7 23318493

[18] Kaptein MJ, Kaptein EM. Inferior Vena Cava collapsibility index: clinical validation and application for assessment of relative intravascular volume. Adv Chronic Kidney Dis. 2021;28(3):218–26. 10.1053/j.ackd.2021.02.003. 34906306 10.1053/j.ackd.2021.02.003

[19] Di Nicolò P, Tavazzi G, Nannoni L, Corradi F. Inferior Vena Cava ultrasonography for volume status evaluation: an intriguing promise never fulfilled. J Clin Med. 2023;12:2217. 10.3390/jcm12062217. 36983218 10.3390/jcm12062217 PMC10053997

[20] Furtado S, Reis L. Inferior Vena Cava evaluation in fluid therapy decision making in intensive care: practical implications. Rev Bras Ter Intensiva. 2019;31(2):240–7. 10.5935/0103-507X.20190039. 31271627 10.5935/0103-507X.20190039 PMC6649212

[21] Middleton WD, Robinson KA. Ultrasound assessment of the hepatic vasculature. In: Middleton WD, Kurtz AB, Hertzberg BS, editors. Ultrasound: the requisites. 3rd ed. Philadelphia: Elsevier; 2015. pp. 233–52.

[22] Alday-Ramírez SM, Leal-Villarreal MAJ, Gómez-Rodríguez C, Abu-Naeima E, Solis-Huerta F, Gamba G, BaezaHerrera LA, Araiza-Garaygordobil D, Argaiz ER. Portal vein doppler tracks volume status in patients with severe tricuspid regurgitation: A proof-of-concept study. Eur Heart J Acute Cardiovasc Care. 2024;13:570–4. 38734970 10.1093/ehjacc/zuae057

[23] Denault AY, Beaubien-Souligny W, Elmi-Sarabi M, Eljaiek R, ElHamamsy I, Lamarche Y, Chronopoulos A, Lambert J, Bouchard J, Desjardins G. Clinical significance of portal hypertension diagnosed with bedside ultrasound after cardiac surgery. Anesth Analg. 2017;124:1109–15. 28151822. 28151822 10.1213/ANE.0000000000001812

[24] Safadi S, Murthi S, Kashani KB. Use of ultrasound to assess hemodynamics in acutely ill patients. Kidney360. 2021;2(8):1349–59. 10.34067/kid.0002322021. 35369668 10.34067/KID.0002322021 PMC8676393

[25] Husain-Syed F, Birk HW, Ronco C, Schormann T, Tello K, Richter MJ, et al. Doppler-derived renal venous stasis index in the prognosis of right heart failure. J Am Heart Assoc. 2019;8(21):e013584. 31630601 10.1161/JAHA.119.013584 PMC6898799

[26] de la Espriella R, Núñez-Marín G, Cobo M, et al. Intrarenal venous flow pattern changes do relate with renal function alterations in acute heart failure. JACC Heart Fail. 2024;12(2):304–18. 10.1016/j.jchf.2023.07.015. 37676214 10.1016/j.jchf.2023.07.015

[27] Beaubien-Souligny W, Benkreira A, Robillard P, Bouabdallaoui N, Chasse M, Desjardins G, et al. Alterations in portal vein flow and intrarenal venous flow are associated with acute kidney injury after cardiac surgery: A prospective observational cohort study. J Am Heart Assoc. 2018;7(19):e009961. 10.1161/JAHA.118.009961. 30371304 10.1161/JAHA.118.009961 PMC6404886

[28] 1Koratala A, Ronco C, Kazory A. Venous doppler ultrasound to guide ultrafiltration in hemodialysis: practical insights for nephrologists. Blood Purif. 2025;54(3):195–9. 10.1159/000543036. 39657602 10.1159/000543036

[29] 2Ataş İ, Yazıcı M, Hamdioğlu E et al. January 15,. (2025) Ultrasonographic Evaluation of Hypervolemic and Normovolemic Patients: A Comparison of Inferior Vena Cava, Subclavian Vein, Internal Jugular Vein, and Femoral Vein Diameters and Collapsibility Indices. Cureus 17(1): e77488. 10.7759/cureus.77488 10.7759/cureus.77488 PMC11827922 39958133

[30] 3Bhardwaj V, Rola P, Denault A, et al. Femoral vein pulsatility: a simple tool for venous congestion assessment. Ultrasound J. 2023;15:24. 10.1186/s13089-023-00321-w. 37165284 10.1186/s13089-023-00321-w PMC10172460

[31] 4Pellicori P, Kallvikbacka-Bennett A, Zhang J, Khaleva O, Warden J, Clark AL, Cleland JG. Revisiting a classical clinical sign: jugular venous ultrasound. Int J Cardiol. 2014;170:364–70. 24315339 10.1016/j.ijcard.2013.11.015

[32] 5Pellicori P, Kallvikbacka-Bennett A, Dierckx R, Zhang J, Putzu P, Cuthbert J, Boyalla V, Shoaib A, Clark AL, Cleland JG. Prognostic significance of ultrasound-assessed jugular vein distensibility in heart failure. Heart. 2015;101:1149–58. 26006717 10.1136/heartjnl-2015-307558

[33] 6Simon MA, Schnatz RG, Romeo JD, Pacella JJ. Bedside ultrasound assessment of jugular venous compliance as a potential Point-of-Care method to predict acute decompensated heart failure 30-Day readmission. J Am Heart Assoc. 2018;7(15):e008184. 10.1161/JAHA.117.008184. 30371245 10.1161/JAHA.117.008184 PMC6201476

[34] 7Parenti N, Palazzi C, Parenti M, D’Addato S. Could internal jugular vein ultrasound be useful in the assessment of patients with heart failure? A systematic review. Italian J Med. 2024;18(2). 10.4081/itjm.2024.1726.

[35] Beaubien-Souligny W, Rola P, Haycock K, et al. Quantifying systemic congestion with Point-Of-Care ultrasound: development of the venous excess ultrasound grading system. Ultrasound J. 2020;12:16. 10.1186/s13089-020-00163-w. 32270297 10.1186/s13089-020-00163-w PMC7142196

[36] Jefkins M, Chan B. Hepatic and portal vein dopplers in the clinical management of patients with right-sided heart failure: two case reports. Ultrasound J. 2019;11(1):30. 10.1186/s13089-019-0146-3. 31748951 10.1186/s13089-019-0146-3 PMC6868079

[37] Rola P, Miralles-Aguiar F, Argaiz E, et al. Clinical applications of the venous excess ultrasound (VExUS) score: conceptual review and case series. Ultrasound J. 2021;13(1):32. 10.1186/s13089-021-00232-8. 34146184 10.1186/s13089-021-00232-8 PMC8214649

[38] Abu-Naeima E, Fatthy M, Shalaby MAAS, et al. Venous excess doppler ultrasound assessment and loop diuretic efficiency in acute cardiorenal syndrome. BMC Nephrol. 2025;26:157. 10.1186/s12882-025-04060-z. 40148759 10.1186/s12882-025-04060-z PMC11951500

[39] Rihl MF, Pellegrini JAS, Boniatti MM. VExUS score in the management of patients with acute kidney injury in the intensive care unit: AKIVEX study. J Ultrasound Med. 2023;42(11):2547–56. 10.1002/jum.16288. 37310104 10.1002/jum.16288

[40] Viana-Rojas JA, Argaiz E, Robles-Ledesma M, et al. Venous excess ultrasound score and acute kidney injury in patients with acute coronary syndrome. Eur Heart J Acute Cardiovasc Care. 2023;12(7):413–9. 10.1093/ehjacc/zuad048. 37154067 10.1093/ehjacc/zuad048

[41] Longino A, Martin K, Leyba K, et al. Prospective evaluation of venous excess ultrasound for Estimation of venous congestion. Chest. 2024;165(3):590–600. 10.1016/j.chest.2023.09.02. 37813180 10.1016/j.chest.2023.09.029 PMC11317813

[42] Albaeni A, Sharma M, Ahmad M, Khalife WI. Accurate Estimation of Right-Filling pressure using handheld ultrasound score in patients with heart failure. Am J Med. 2022;135:634–40. 34979092 10.1016/j.amjmed.2021.11.020

[43] Torres-Arrese M, Mata-Martínez A, Luordo-Tedesco D, García-Casasola G, Alonso-González R, Montero-Hernández E, Cobo-Marcos M, Sánchez-Sauce B, Cuervas-Mons V, Tung-Chen Y. The usefulness of systemic venous ultrasound protocols in the prognosis of heart failure patients: results from a prospective multicentric study. J Clin Med. 2023;12:1281. 36835816 10.3390/jcm12041281 PMC9966251

[44] Sovetova S, Charaya K, Erdniev T, Shchekochikhin D, Bogdanova A, Panov S, Plaksina N, Mutalieva E, Ananicheva N, Fomin V, et al. Venous excess ultrasound score is associated with worsening renal function and reduced natriuretic response in patients with acute heart failure. J Clin Med. 2024;13:6272. 39458220 10.3390/jcm13206272 PMC11508279

[45] Beaubien-Souligny W, Galarza L, Buchannan B, Lau VI, Adhikari NKJ, Deschamps J, Charbonney E, Denault A, Wald R. Prospective study of ultrasound markers of organ congestion in critically ill patients with acute kidney injury. Kidney Int Rep. 2023;9:694–702. 38481488 10.1016/j.ekir.2023.12.018 PMC10927464

[46] Prager R, Argaiz E, Pratte M, et al. Doppler identified venous congestion in septic shock: protocol for an international, multi-centre prospective cohort study (Andromeda-VEXUS). BMJ Open. 2023;13(7):e074843. 10.1136/bmjopen-2023-074843. 37487682 10.1136/bmjopen-2023-074843 PMC10373747

[47] Romano M, Viana E, Martins JD, Corga Da Silva R. Evaluation of congestion levels in septic patients admitted to critical care units with a combined venous Excess-Lung ultrasound score (VExLUS) - a research protocol. POCUS J. 2023;8(1):93–8. 10.24908/pocus.v8i1.16188. 37152345 10.24908/pocus.v8i1.16188 PMC10155730

[48] Balci A, Karazincir S, Sumbas H, Oter Y, Egilmez E, Inandi T. Effects of diffuse fatty infiltration of the liver on portal vein flow hemodynamics. J Clin Ultrasound. 2008;36(3):134–40. 10.1002/jcu.20440. 18196595 10.1002/jcu.20440

[49] Schneider AW, Kalk JF, Klein CP. Hepatic arterial pulsatility index in cirrhosis: correlation with portal pressure. J Hepatol. 1999;30:876–81. 10365815 10.1016/s0168-8278(99)80142-1

[50] Sakai K, Nakamura K, Satomi G, Kondo M, Hirosawa K. Evaluation of tricuspid regurgitation by blood flow pattern in the hepatic vein using pulsed doppler technique. Am Heart J. 1984;108(Pt 1):516–23. 6475714 10.1016/0002-8703(84)90417-4

